# Preventive effect of teriparatide on medication-related osteonecrosis of the jaw in rats

**DOI:** 10.1038/s41598-023-42607-y

**Published:** 2023-09-19

**Authors:** Kyeong-Mee Park, Namkwon Lee, Jaeyeon Kim, Hyun Sil Kim, Wonse Park

**Affiliations:** 1 Department of Advanced General Dentistry, Human Identification Research Institute, Yonsei University College of Dentistry, Seoul, Republic of Korea; 2 Department of Advanced General Dentistry, Yonsei University College of Dentistry, Seoul, Republic of Korea; 3 Department of Oral Pathology, Oral Cancer Research Institute, Yonsei University College of Dentistry, Seoul, Republic of Korea

**Keywords:** Microbiology, Diseases

## Abstract

This study aimed to investigate the preventive effect of teriparatide (TPD) administration on medication-related osteonecrosis of the jaw (MRONJ) before tooth extraction due to periodontal lesions in bilaterally ovariectomized female rats treated with zoledronic acid. Thirty skeletally mature Sprague–Dawley rats were randomly divided into three groups: control (CONT, n = 10), zoledronic acid (ZA, n = 10), and zoledronic acid and teriparatide (ZA-TPD, n = 10). The rats were sacrificed 8 weeks after tooth extraction. Micro-computed tomography analysis of the tibia showed that bone mineral density was highest in the CONT, followed by that in the ZA and ZA-TPD groups (CONT/ZA, *p* = 0.009; CONT/ZA-TPD, *p* < 0.001; ZA/ZA-TPD, *p* < 0.001). In the trabecular bone analysis of the extraction site, significant differences in specific bone surface (CONT/ZA, *p* = 0.010; CONT/ZA-TPD, *p* = 0.007; ZA/ZA-TPD, *p* = 0.002) and trabecular thickness (CONT/ZA-TPD, *p* = 0.002; ZA/ZA-TPD, *p* = 0.002) were observed. Histological analyses of the extraction sites revealed characteristic MRONJ lesions in the ZA group. Osteonecrosis, inflammatory cells, and sequestrum were less frequently observed in the ZA-TPD group than in the ZA group. In conclusion, TPD administration before tooth extraction helped reduce the occurrence of MRONJ in rats treated with zoledronic acid, confirming its preventative effects.

## Introduction

Bisphosphonate (BP) is an anti-resorptive agent for the prevention of bone density loss in patients with osteoporosis. Bone tissue helps maintain homeostasis by continuously forming and breaking down bone using osteoblasts and osteoclasts, respectively. BP prevents bone loss by inducing osteoclast apoptosis^1^. Thus, it is recommended as a first-line treatment for postmenopausal osteoporosis, effective in preventing fractures with long-term use^2,3^. Zoledronic acid (ZA), used for intravenous therapy in high-risk of fracture groups, and significantly reduces the risk of hip and vertebral fractures^4^. However, despite the various benefits of BP, MRONJ is known as a potential side effect of anti-resoprtive agents^5^.

MRONJ is an expanded concept of bisphosphonate-related osteonecrosis of the jaw and diagnosed when certain criteria are present^6^. Notably, MRONJ is a rare multifactorial disease that involves both inflammation and infection^6^. Various animal studies have been conducted to understand the pathophysiology of MRONJ^7,8^. However, most of these studies did not properly reflect the clinical environment by conducting experiments in healthy rats without systemic disease or extracting healthy teeth after BP administration^7,8^.

Bone necrosis associated with BP occurs only in the jawbone due to its predisposition to bacterial infections^9,10^. It is known that the main factor triggering MRONJ is invasive treatment such as tooth extraction, but it is argued that bacterial infection, not invasive treatment itself, is the main risk factor ^11^. Consequently, the importance of preventive treatment, such as removing inflammation before dental treatment, has been emphasized^12^. Most EXTs are performed to treat inflammatory lesions such as periodontitis^6^. Osteonecrosis of the jaw (ONJ) has been shown to be aggravated by bacterial infections; however, its incidence decreased with oral hygiene improvement^13^. This suggests that bacterial infections play an important role in the development of MRONJ.

Treating underlying dental conditions before the use of BP is the key in preventing MRONJ. However, if a patient is already taking BP and requires invasive dental treatment, a withdrawal period may be considered in consultation with their physician. However, according to a recent American Association of Oral and Maxillofacial Surgeons (AAOMS) position paper, drug holidays are controversial^6^. Although various prevention and treatment methods have been proposed for MRONJ, no gold standard has been established^6^. Since osteoclast activation is suppressed by BP in MRONJ, TPD treatment, which can stimulate osteoclast activity, is effective in preventing MRONJ^14,15^. Intermittent TPD can increase new bone formation, bone mass, bone density, and bone quality through anabolic effects^16^.

Although bacterial infection is a factor in the development of MRONJ, no animal model accurately reflected the clinical environment. Therefore, research is needed to develop an animal model that accurately reflects the clinical environment prior to the development of a standard treatment for MRONJ prevention. In this study, it was expected that TPD would reduce the risk of MRONJ by promoting alveolar bone healing. The null hypothesis of this study was that TPD has no preventive effect on the development of MRONJ. To confirm this hypothesis, the MRONJ rat model in which ZA was administered before tooth extraction was used.

## Results

### Animals

Evaluations related to animals is described in the Supplementary Appendix.

### Proximal tibia: micro-CT

**Figure 1:**
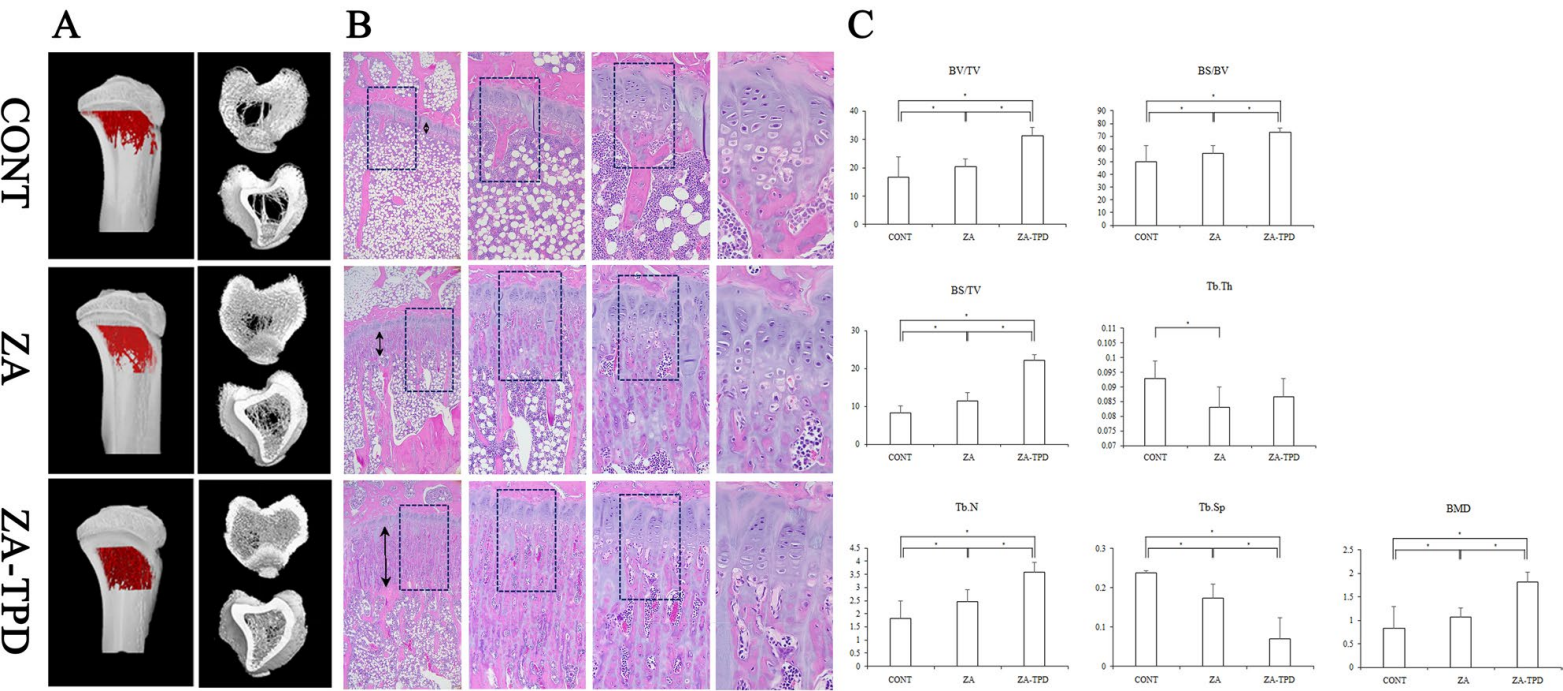
Evaluation of the systemic effect of zoledronic acid and teriparatide in the proximal tibia. Teriparatide increases the bone density of the proximal tibia and improves bone quality. (**A**) Three-dimensional cross-sectional images using micro-CT (lateral view, top view, bottom view). Trabecular bone was the least amount in the CONT group and most amount in the ZA-TPD group. (**B**) Histological images of epiphyseal cartilage (40, 100, 200, 400 magnification). Primary spongiosa showed a large difference among the groups and gradually thickened in the following order: CONT, ZA, and ZA-TPD. (**C**) Three-dimensional analysis of trabecular bone using micro-CT. Black double arrow indicates primary spongiosa zone. Asterisk (*) means statistically significant (*P* < 0.017). *CT* computed tomography, *CONT* control, *ZA* zoledronic acid, *ZA-TPD* zoledronic acid and teriparatide.

The ZA-TPD group showed the greatest increase in trabecular bone microstructure, followed by the ZA and CONT groups, respectively (Fig. 1A).

Quantitative analysis showed that BV/TV, BS/BV, BS/TV, Tb.N, and BMD significantly increased in the ZA-TPD group compared with the other groups (*p* < 0.001) and in the ZA group compared to the CONT group (*p* = 0.009). Conversely, Tb.Sp significantly decreased in the ZA-TPD group compared with the other groups (*p* < 0.001) and in the ZA group compared with the CONT group (*p* = 0.002). Additionally, Tb.Th was highest in the CONT group and decreased in the ZA and ZA-TPD groups, respectively; however, a statistically significant difference was only observed between the CONT and ZA groups (*p* = 0.006) (Fig. 1C).

### Proximal tibia: histology

Differences in the thickness of the growth plates of the proximal tibia were not observed among the groups. However, the proliferative zone of the CONT group could be more clearly observed compared with that of the other groups (Fig. 1B). In contrast, the thickness of the primary spongiosa showed a large difference among the groups, with gradual thickening observed in the following order: CONT, ZA, and ZA-TPD. Additionally, the primary spongiosa was barely observed in the CONT group but remarkably thickened in the ZA-TPD group (Fig. 1B).

### EXT socket: micro-CT

**Figure 2:**
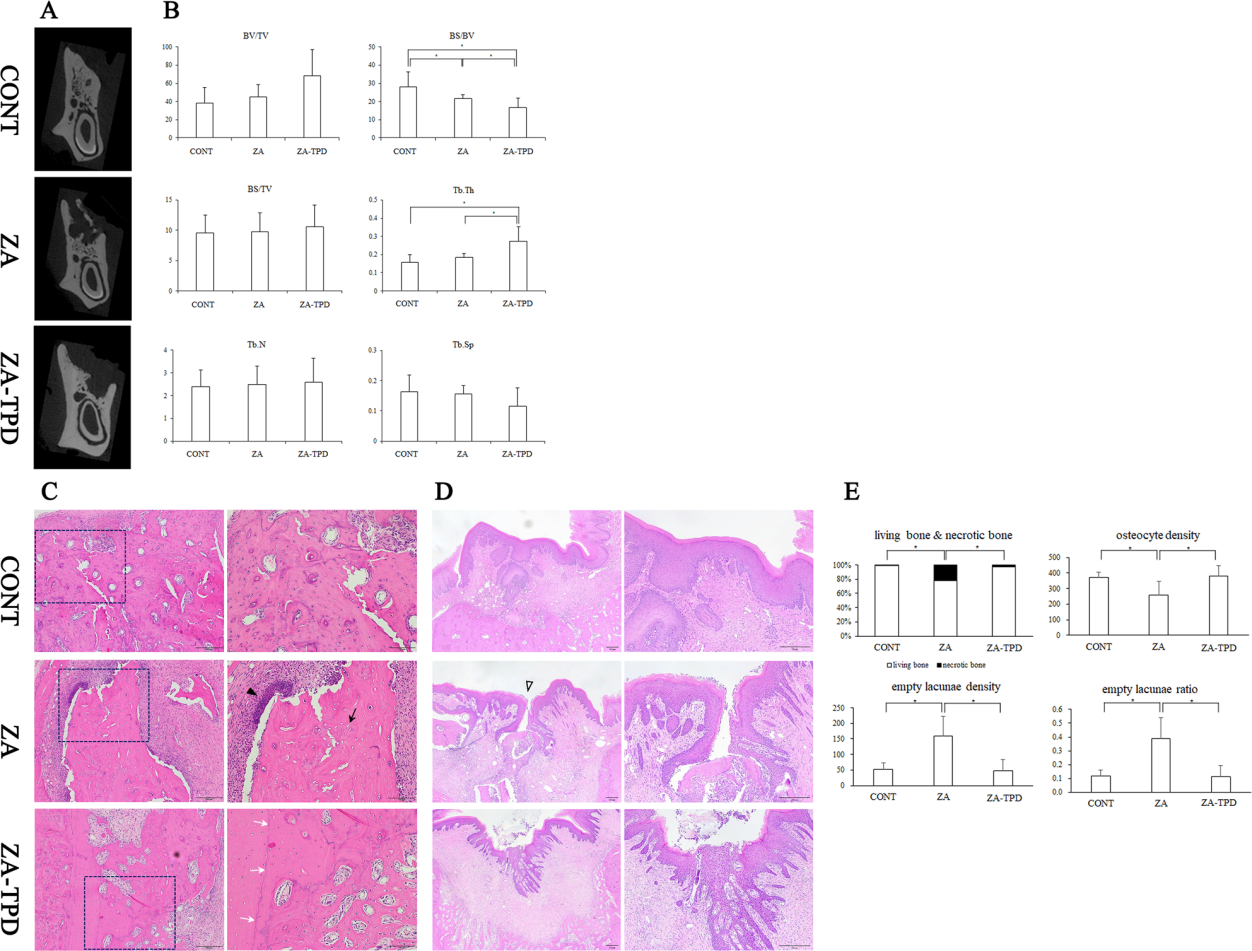
Comparison of changes in extraction site following systemic administration of zoledronic acid and teriparatide. TPD induced healing of the extraction socket. (**A**) Two-dimensional coronal plane images using micro-CT. TPD induced healing of the extraction socket as much as in the CONT group. (**B**) Three-dimensional analysis using micro-CT. (**C**) Bone around extraction sockets on H-E staining (100, 200 magnification). In the ZA group, sequestrum, empty lacunae, and inflammatory cells were observed in all samples, and in the TPD group, extraction socket healing was observed. (**D**) Epithelium images around extraction socket on H-E staining (40, 100 magnification). (**E**) Histomorphometric analysis with H-E staining. Black arrowhead indicates inflammatory cell. Black arrow indicates empty lacunae. White arrow indicates reversal line. White arrowhead indicates opened wound. Asterisk (*) means statistically significant (P < 0.017). *CT* computed tomography, *H–E* hematoxylin and eosin, *CONT* control, *ZA* zoledronic acid, *ZA-TPD* zoledronic acid and teriparatide.

In the two-dimensional cross-sectional radiographic image of the EXT socket (Fig. 2A), the EXT socket healed well in the CONT group. In contrast, bone healing was incomplete at the EXT site in the ZA and ZA-TPD groups, and sequestrum was observed in the ZA group. The density of trabecular and cortical bone was higher in the ZA-TPD group than in other groups.

Three-dimensional microarchitecture analysis of the trabecular bone showed that BV/TV, BS/TV, Tb.Th, and Tb.N were in the least amount in the CONT group and increased the most in the ZA-TPD group (Fig. 2B). Among these, only Tb.Th significantly increased in the ZA-TPD group compared with the other groups (*p* = 0.002). In contrast, BS/BV and Tb.Sp decreased the most in the ZA-TPD group, followed by the ZA and ZA-TPD groups. Only BS/BV showed statistically significant differences between the groups (CONT/ZA, *p* = 0.010; CONT/ZA-TPD, *p* < 0.007; ZA/TPD, *p* < 0.002).

### EXT socket: histology

The EXT socket of the CONT group showed normal healing with no abnormal findings such as bone exposure (Fig. 2C). In the ZA group, bone healing at the extraction site was incomplete, and exposed bones were observed in three specimens (Fig. 2D). Additionally, sequestrum, empty lacunae, and inflammatory cells were observed in all specimens (Fig. 2C). In the ZA-TPD group, necrotic bone was observed in only one specimen, and exposed bone was observed in three specimens. Moreover, inflammatory and sequestered cells and reversal lines were clearly observed in most samples (Fig. 2C).

### EXT socket: histomorphometry analysis

Histomorphometric analysis results obtained from the EXT sites of all specimens indicated that the living bone of the ZA group was significantly less than that of the CONT (*p* = 0.002) and ZA-TPD (*p* = 0.006) groups, whereas the number of necrotic bones was significantly higher (CONT/ZA, *p* = 0.002; ZA/ZA-TPD, *p* = 0.006) (Fig. 2E). Osteocyte density was significantly lower in the ZA group than in the CONT (*p* = 0.002) and ZA-TPD (*p* = 0.002) groups, whereas empty lacunae density (CONT/ZA, *p* < 0.001; ZA/ZA-TPD, *p* < 0.001) and empty lacunae ratio (CONT/ZA, *p* < 0.001; ZA/ZA-TPD, *p* < 0.001) were significantly higher in the ZA group.

### qPCR analysis

**Figure 3:**
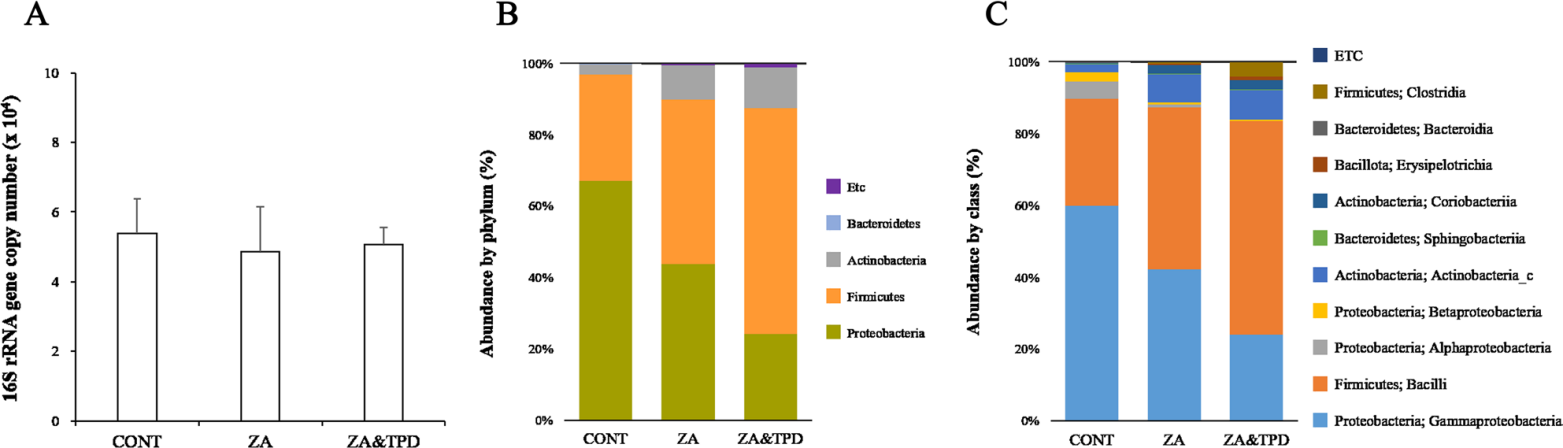
Oral microbiota composition appeared equally in all groups. (**A**) Firmicutes (Bacilli, Clostridia). (**B**) Proteobacteria (α-proteobacteria, β-proteobacteria, γ-proteobacteria). (**C**) Class level.

The total load of oral microbiota collected from the silk was uniform in all groups (Fig. 3A) and have a highly diverse composition (Fig. 3B, C). Proteobacteria and Firmicutes accounted for most of the microorganisms in all groups (Fig. 3B). The composition of Proteobacteria and Bacteroidetes decreased in the following order: CONT, ZA, and ZA-TPD groups. Meanwhile, Firmicutes and Actinobacteria gradually increased in the following order: CONT, ZA, and ZA-TPD groups (Fig. 3B). γ-proteobacteria and bacilli were dominant in all groups (Fig. 3C). However, there were no statistically significant differences between the bacterial population in all groups overall.

## Discussion

The null hypothesis of this study was that TPD has no preventive effect on the development of MRONJ. To confirm this hypothesis, the MRONJ rat model in which ZA was administered before tooth extraction was used.

The most likely cause of MRONJ is a mechanism in which dysfunction of osteoclasts and osteoblasts, along with bacterial infection, leads to a decrease in bone remodeling^6^. In this study, the occurrence of MRONJ increased when EXT was performed after ZA administration in rats with periodontitis; however, short-term administration of TPD before EXT decreased the incidence of MRONJ. Furthermore, systemic bone quality and BMD improved when TPD was administered.

Rodgers et al.^17^ reported that convenience, relevance, and suitability should be considered when selecting a model suitable for osteoporosis. Rats are widely used as an osteoporosis model because of the ease in handling them and their calcium metabolism similar to humans^18^. However, since rats often do not have menopause, OVX is used as a means to induce menopause^19^. They are also known to mature sexually and skeletally at 2.5 and 10 months, respectively^20,21^. Rodents are highly reproducible when inducing periodontitis and are easier to handle than other animals^22–24^. The periodontitis animal model is classified into a ligation-induced model and pathogenic bacterial infusion model. The ligation-induced model is commonly used because it can reproduce an environment similar to that in human periodontitis^25^. In the case of a model injected with pathogenic bacteria, periodontitis is induced by directly injecting lipopolysaccharide subgingivally^26,27^. The bacterial injection model has the disadvantage of low reproducibility of periodontitis and being time-consuming compared to the ligation model. Therefore, we performed OVX at 12 weeks of age, which is the first maturation period, and reproduced MRONJ using a ligation-induced periodontitis model in an osteoporosis-induced rat model^28^.

Although controversies regarding the treatment method for MRONJ remain, intermittent parathyroid hormone treatment is attracting attention as an adjuvant therapy in addition to surgical procedures^29,30^. TPD is a recombinant protein of parathyroid hormone, an agent that acts directly on bone tissue and is an 84-amino-acid chain. TPD consists of 34 amino-terminals and has high sequence conservation in rats and humans; hence, the experiment was conducted in rats^31^. Moreover, because mice and humans show similar calcium metabolism^18^, they are most often used as animal models for bone metabolic disease studies^16,32^. In this study, radiological and histological evaluations were performed on the proximal tibia to evaluate the systemic effect of the drug. Results showed that ZA and TPD significantly increased bone quality (Fig. 1–2). TPD (20–200 μg/kg) is used in various ways for fracture treatment, MRONJ management, and bone transplantation^16,33,34^. Moreover, it was most effective clinically and histologically for MRONJ treatment when administered at 20 μg/kg^34^. The systemic effect of TPD was confirmed in the proximal tibia as bone quality improved both radiographically and histologically (Fig. 1). Histological observations showed that TPD administration activated the development of the primary spongiosa zone below the growth plate, suggesting that the mineralization process in the cartilage is active and may further promote ossification activity. Furthermore, the administration of TPD 2 weeks before EXT is effective in preventing ONJ ^35^. Similar to the results of these study, when only ZA was administered at the EXT site, necrotic bone increased in an MRONJ-like state; however, when TPD was administered before EXT, the amount of necrotic bone was at the same level as that in the CONT group (Fig. 2).

The proximal tibia was used to observe the systemic effects of ZA and TPD using micro-CT analysis (Fig. 1A). When only ZA was administered, BV/TV, Tb.N, and BMD increased. When TPD was additionally administered, BV/TV, Tb.N, and BMD increased significantly (Fig. 1C). Both BS/BV and BS/TV increased in the experimental group; however, Tb.Sp significantly decreased (Fig. 1C). These results indicate that the systemic administration of ZA and TPD improved osteoporotic status in osteoporosis-induced rats, which is consistent with the results of previous studies^34,35^. The appropriate dose and usage of ZA and TPD was then determined. However, in the mandible, BS/BV was highest in the CONT group and lowest in the ZA-TPD group (Fig. 2B). Conversely, Tb.Th was highest in the ZA-TPD group, and a statistically significant difference was found only when compared with the ZA-TPD group (Fig. 2B). This means that the bone quality of the ZA-TPD group is the best. In previous animal experiments, there was a large difference in the analytical method of each experiment, such as the nonperformance of micro-CT analysis in the EXT socket^34,35^ or different ROI locations within the EXT socket^36,37^. This may be due to the difficulty of setting ROIs, as various anatomical structures are located around the experimental site, and that histological findings are more meaningful than radiological findings for the evaluation of MRONJ. These problems are may have affected the micro-CT results of the mandible in our study.

Histological evaluation of MRONJ has been conducted in many studies and may be considered as a basic step in understanding the mechanism of MRONJ. In general, histologically evaluating the state of bone healing, epithelial integrity, inflammation, necrotic bone, osteoblasts, osteoclasts, and blood vessels are used as standard indicators^38^. In our study, inflammation, necrotic bone, sequestrum, and exposed bone, which are the most important features for MRONJ development, were evaluated. In some studies, inflammatory cells were divided into four stages and classified^39,40^. In our study, only the presence/absence of inflammation was evaluated without classification. Inflammation was observed in 100% of the ZA group, 80% of the ZA-TPD group, and 40% of the CONT group (Fig. 2C). In our experiment, root rest was confirmed in two cases for CONT, one case for ZA, and two cases for TPD. The observed inflammation of 40% in CONT is thought to be influenced by root rest. Necrotic bone is the most representative feature of MRONJ; however, the diagnostic criteria for necrotic bone are very diverse. In our study, it was defined as a devital bone area with 10 or more adjacent empty lacunae^41^. The amount of necrotic bone was significantly higher in the ZA group, and the ZA-TPD group has an amount similar with that in the CONT group (Fig. 2E). Sequestrum was observed in all specimens in the ZA group, but only in seven specimens in the ZA-TPD group. Meanwhile, bone exposure was confirmed in two and three specimens in the ZA and ZA-TPD groups, respectively, and no difference was observed between the two groups. Based on these histological findings, it is speculated that during the process of inducing MRONJ by ZA, TPD induces osteoblast activity and promotes bone modeling to prevent MRONJ occurrence.

Studies have been conducted on various factors known to increase the risk of MRONJ, such as the type of drug used, method of administration, duration of administration, and dental diseases^7,8^. Clinically, more severe periodontitis or periapical diseases are treated with EXT, which increases the risk of MRONJ. MRONJ is a disease caused by various factors, including anti-resorptive medication, inflammation, and infection^6^. Therefore, in this study, MRONJ was induced by extraction after inducing periodontitis through tooth ligation. Most animal models of periodontitis are made by ligating silk into the tooth^42^. In some studies, animal models of periodontitis have been made by injecting lipopolysaccharide^7^ or bacterial pathogens^42^ into the gingival fissure.

According to Masa et al.^43^, the total amount of bacterial DNA collected from periodontal lesions of IL-17 KO mice, which plays an important role in host defense against bacterial infection, was significantly increased compared to that in wild-type mice. We created an animal model in which periodontitis was induced using silk^22^, and oral bacteria that accumulated around the silk caused inflammation, leading to alveolar bone destruction. To confirm that periodontitis was induced, oral microbiota from the collected silk was analyzed. Equal amounts of microbiota were detected in all groups (Fig. 3A), and there was no statistical difference in the number and distribution of Bacteroides and Firmicutes associated with periodontitis. Moreover, Proteobacteria and Actinobacteria, which are often observed in healthy environments without inflammatory findings, also showed no statistical differences in the quantity and distribution rate detected. A previous study reported a significant increase in the distribution of γ-proteobacteria in KO mice^43^. In our study, in addition to Bacteroidia, Clostridia, Bacilli, and Sphingobacteriia, which are associated with periodontitis, we also found oral bacterial, such as α-proteobacteria, β-proteobacteria, γ-proteobacteria, Actinobacteria_c, and coriobacteriia, which are mainly observed in healthy environments. These populations did not differ between groups, suggesting that periodontitis was well-induced in all groups, and that MRONJ was difficult to develop with only periodontitis.

## Conclusion

On the other hand, a limitation of our study is that we did not set up a control group in which periodontitis was not induced. Additional studies are needed to evaluate the contribution of the oral microbiota to the occurrence and prevention of MRONJ in a periodontitis-free environment and determine the effective dose and duration of TPD to confirm its preventive effect on MRONJ. In conclusion, TPD administration before EXT is effective in preventing MRONJ.

## Methods

### Animals

Animal selection, management, surgical protocol followed the protocols approved by the Institutional Animal Care and Use Committee of Yonsei Medical Center (IACUC No. 2018-0210) and conformed to the ARRIVE 2.0 guidelines. More details are described in the Appendix. All the methods in this study were performed in accordance with relevant guidelines and regulation.

### Experimental design

The rats were randomly divided into three groups: control (CONT, n = 10), zoledronic acid (ZA, n = 10), and zoledronic acid and teriparatide (ZA-TPD, n = 10). Sample size was determined with an effect size of 0.9 and power of 0.7. A total sample size of 26 was calculated. Finally, a total of 30 samples was employed, 10 per group, considering a dropout rate of 15%. The effect size was calculated by referring to data of a previous study^34^. Bilateral ovariectomy (OVX) was performed to induce osteoporosis.

Body weight was measured weekly before OVX until the end of the experiment. The rats were subjected to bilateral OVX to induce osteoporosis. Eight weeks later, periodontal lesions (mandibular 2nd molar) were induced via silk ligation.

Four weeks after the ligation, the ZA and ZA-TPD groups were administered with ZA (Zometa; Novartis, Basel, Switzerland) through weekly intravenous injection for 4 weeks (40 μg/kg × day). The ZA-TPD group was then administered with TPD (Forsteo; Eli Lilly, Houten, The Netherlands) via subcutaneous injection every day for 4 weeks (20 μg/kg × day). During the same period, the CONT and ZA groups (sham-ZA and sham-TPD) received injections of the same volume and method of saline. Ten weeks after the ligation, the ligated teeth were extracted. The rats were sacrificed 8 weeks after EXT (Supplementary Fig. S1). More details are described in the Supplementary Appendix.

### Micro-computed tomography(micro-CT)

Micro-CT (skyscan1173; SkyScan, Kontich, Belgium) was performed on the tibia and mandible (7.1, 12.07 µm), which were also analyzed using CTAn software (CTAn; SkyScan, Kontich, Belgium) to evaluate the systemic effects of the drug. The region of interest (ROI) was decided at 0.7 to 3.5 mm below the growth plate to analyze the trabecular bone microstructure of the tibia. The grayscale was set to 30–255 (Supplementary Fig. S2A). The ROIs for the evaluation of the trabecular bone morphometry were set in a cylindrical shape with Ø 0.6 mm and 0.5 mm length, and the grayscale was set to 50–255 (Supplementary Fig. S2B).

The bone volume fraction (BV/TV, %), specific bone surface (BS/BV, mm^2^/mm^3^), bone surface density (BS/TV, mm^2^/mm^3^), trabecular thickness (Tb.Th, mm), trabecular number (Tb.N, 1/mm), trabecular separation (Tb.Sp, mm), and bone mineral density (BMD, g/cm^2^) were used to evaluate the trabecular bone microarchitecture.

### Histology

The specimens were demineralized with 10% ethylene-diamine-tetraacetic acid for 4 weeks. After paraffin embedding, the blocks were stained with hematoxylin and eosin. The stained tissue was observed under an optical microscope (OLYMPUS BX43; Olympus Corporation, Tokyo, Japan). Living bone (%, the bone area with morphologically normal osteocytes), necrotic bone (%, the devital bone area in which there were ≥ 10 pyknotic osteocyte lacunae), osteocyte density (#/mm^2^, number of osteocytes), empty lacunae density (#/mm^2^, number of empty osteocyte lacunae), and empty lacunae ratio (number of empty lacunae per total number of osteocyte lacunae) were quantified in the mandible specimens using  200× magnified images from above the EXT site.

### Quantitative polymerase chain reaction (qPCR) analysis: oral microbiota

Silk ligation was maintained for 10 weeks. Bacterial DNA was extracted using a kit (FastDNA Spin Kit; MP Biomedicals, Solon, OH, USA). The primer sequences used were: F341 ‘CCTACGGGNGGCWGCAG’ and R805 ‘GACTACHVGGGTATCTAATCC’.

### Statistical analysis

Statistical analyses were performed using IBM SPSS Statistics for Windows (Version 25; IBM, Armonk, NY, USA). After the Kruskal–Wallis test, the three groups were compared using the post-hoc Mann–Whitney U test. Statistical significance was set at *p* < 0.017^44^.

### Supplementary Information

Supplementary Information.

Supplementary Figures.

## Data Availability

The data that support the findings of this study are available from the corresponding author upon reasonable request.
