# Supplementary material for: Preventive effect of teriparatide on medication-related osteonecrosis of the jaw in rats
Source: Sci Rep. 2023 Sep 19;13:15518. doi: 10.1038/s41598-023-42607-y (PMC10509150; doi:10.1038/s41598-023-42607-y)
Supplement: Supplementary file 1 — Supplementary Information. [file 41598_2023_42607_MOESM1_ESM.docx]

**Preventive effect of teriparatide on medication-related osteonecrosis of the jaw in rats**

Kyeong-Mee Park^1†^, Namkwon Lee^2†^, Jaeyeon Kim^2^, Hyun Sil Kim^3^, Wonse Park^2*^

**Appendix**

**Materials and Methods**

***Animals***

Thirty skeletally mature 12-week-old 250 g female Sprague-Dawley rats (Orientbio , Suwon, Korea) were used in this study. All animals were housed and experiments were conducted under the supervision of the Laboratory Animals Department of the Avison Medical Life Research Center, Yonsei University College of Medicine. All experimental procedures were conducted in accordance with the accreditation standards of the International Association for Assessment and Accreditation of Laboratory Animal Care. Three animals were housed per cage at a temperature of 20±5°C and humidity of 50%±10%, with a 12-hour light-dark cycle, and a standard laboratory diet provided ad libitum. The animals were acclimatized for one week prior to the commencement of the experiments. Animal selection, management, surgical protocol, and preparation followed routine protocols approved by the Institutional Animal Care and Use Committee of Yonsei Medical Center, Seoul, Korea (IACUC No. 2018-0210).

***Experimental design***

The sample size was established based on the results of our pilot study. Body weight was measured every week from before ovariectomy until the end of the experiment. All animals were subjected to bilateral ovariectomy under general anesthesia to induce osteoporosis. General anesthesia was performed by intraperitoneal injection of combined tiletamine and zolazepam (Zoletil; Virbac lab, Carros, France [50mg/ml, 0.6ml/kg]) and xylazine (Rompun; Bayer, Leverkusen, Germany [23.32 mg/ml, 0.4 ml/kg]). For relief of infection and pain after OVX, meloxicam (Metacam; Boehringer Ingelheim, Rhein, Germany [1 mg/kg, once a day for 5 days]) and enfloxacin (Baytril; Bayer, Leverkusen, Germany [10 mg/kg, once a day for 5 days]) was administered subcutaneously.

Eight weeks post ovariectomy, after general anesthesia, local anesthesia (2% lidocaine hydrochloride with 1: 80,000 epinephrine) was administered, and periodontitis was induced by ligation of the mandibular second molar with 4-0 black silk (Mersilk; Ethicon, New Jersey, USA). After silk ligation, the knot was tied three times in the linguo-distal direction.

Intensive observation was conducted for one week after surgery to see if any unexpected side effects occurred, and weekly monitoring was performed during the experimental period. The order of treatment and measurement of animals was randomized to minimize potential confounding factors. And all the data analysis was performed in a blind manner by randomly mixing the sample.

**Results**

***Animals***

The average body weight of the rats before OVX was 250 g, and the rate of increase in body weight was constant until the animals were euthanized. All the rats gained weight, with no significant difference in the rate of weight gain. Humane termination of the experiment was not performed because weight loss, behavioral abnormalities, and serious infection at the surgical site were not found.

**Supplementary Figure S1.** Study design. OVX, ovariectomy; Ext., tooth extraction; ZA inj., zoledronic acid injection; s-ZA inj., sham zoledronic acid injection; TPD inj., teriparatide injection; s-TPD inj., sham teriparatide injection.

**Supplementary Figure S2.** The region of interest (ROI) for micro-computed tomography analysis. (**A**) ROI of the proximal tibia. (**B**) ROI of the extraction site in the mandible. (**a**) growth plate. (**b**) mandibular canal. (**c**) incisor root.

***Data access***

The study data is available in the manuscript and appendix submission.
