# Supplementary material for: Preventive effect of teriparatide on medication-related osteonecrosis of the jaw in rats
Source: Sci Rep. 2023 Sep 19;13:15518. doi: 10.1038/s41598-023-42607-y (PMC10509150; doi:10.1038/s41598-023-42607-y)

**Supplementary Figure S1.** Study design. OVX, ovariectomy; Ext., tooth extraction; ZA inj., zoledronic acid injection; s-ZA inj., sham zoledronic acid injection; TPD inj., teriparatide injection; s-TPD inj., sham teriparatide injection.


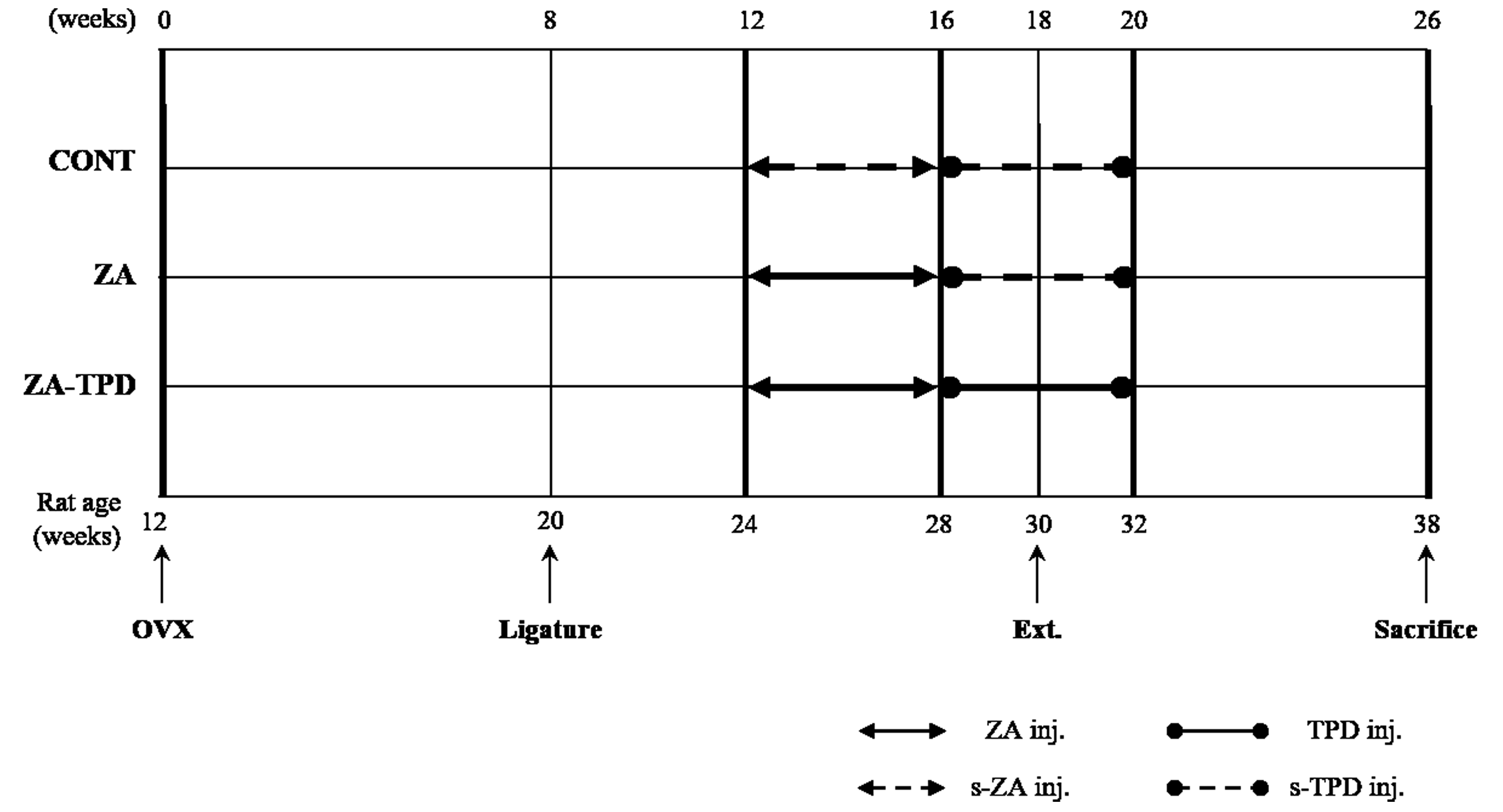


**Supplementary Figure S2.** The region of interest (ROI) for micro-computed tomography analysis. (**A**) ROI of the proximal tibia. (**B**) ROI of the extraction site in the mandible. (**a**) growth plate. (**b**) mandibular canal. (**c**) incisor root.


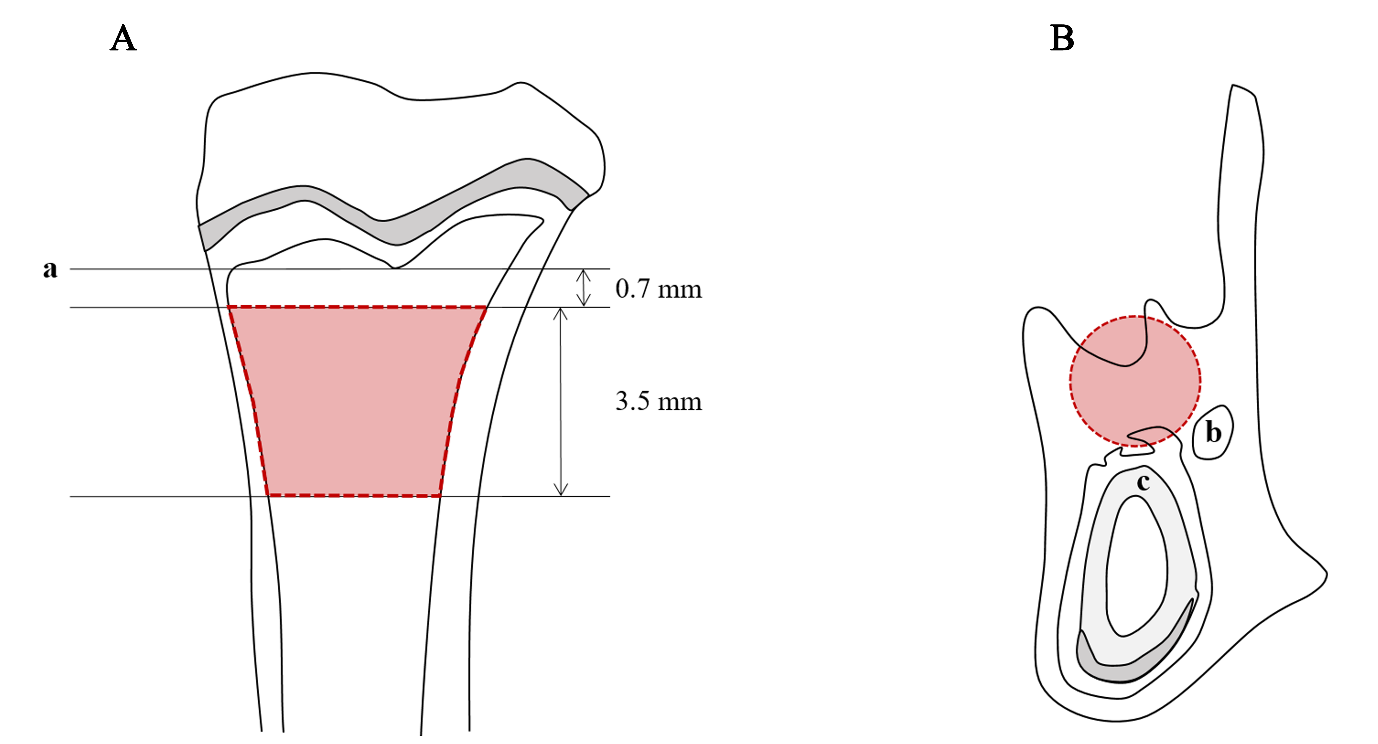

Supplement: Supplementary file 2 — Supplementary Figures. [file 41598_2023_42607_MOESM2_ESM.docx]
